# Real-World Vaccination Data Analysis for Three Vaccines Among Individuals Aged 60 and Older in Shanghai, China

**DOI:** 10.3390/vaccines14030246

**Published:** 2026-03-07

**Authors:** Juan Li, Mingzhu Lin, Yong Feng, Wanran Cheng, Cuiping Zhou, Shaotan Xiao, Pengfei Deng, Laibao Yang

**Affiliations:** Shanghai Pudong New Area Center for Disease Control and Prevention (Shanghai Pudong New Area Health Supervision Institute), Shanghai 200136, China; lijuan15180219@163.com (J.L.); mzlin@pdcdc.sh.cn (M.L.); yongfeng@pdcdc.sh.cn (Y.F.); m13914785746@163.com (W.C.); cpzhou@pdcdc.sh.cn (C.Z.); stxiao@pdcdc.sh.cn (S.X.); masterdpf@163.com (P.D.)

**Keywords:** influenza vaccine, herpes zoster vaccine, 23-valent pneumococcal polysaccharide vaccine, vaccination coverage, older adults

## Abstract

**Background**: Vaccines targeting herpes zoster, influenza, and pneumococcal diseases represent the most effective interventions for reducing morbidity and mortality in individuals aged ≥65 years. This study employs real-world vaccination data for herpes zoster vaccine (HZV), influenza vaccine (InfV), and 23-valent pneumococcal polysaccharide vaccine (PPSV23) among individuals aged ≥60 years in the Pudong New Area of Shanghai, China, from 2020 to 2024, aiming to assess the vaccination coverage for the three vaccines. **Methods**: Demographic data and vaccination records for HZV, InfV, and PPSV23 were obtained from the Shanghai Immunization Information System. Vaccination coverage, temporal trends, and disparities across different demographic groups and subdistricts or towns were analyzed. **Results**: From 2020 to 2024, a total of 26,227 doses of HZV, 198,373 doses of InfV, and 102,644 doses of PPSV23 were administered to adults aged ≥60 years in the Pudong New Area of Shanghai, with vaccination coverage of 0.23%, 3.12%, and 1.61%, respectively. HZV coverage peaked in 2023 (0.34%), whereas the highest coverage for InfV (3.94%) and PPSV23 (3.21%) occurred in 2020. The highest vaccination coverage was observed in the 70–74 age group for HZV (0.30%), the 75–79 age group for InfV (5.18%), and the 65–69 age group for PPSV23 (2.15%). Coverage for HZV and InfV was higher among females than males, while PPSV23 coverage was higher among males. Individuals with local household registration had significantly higher coverage for all three vaccines compared to those with non-local registration. The subdistricts or towns with the highest HZV coverage were Jinqiao Town (0.59%), Huamu Subdistrict (0.50%), and Lujiazui Subdistrict (0.34%). For InfV, the highest coverage was observed in Tangqiao Subdistrict (5.50%), Huamu Subdistrict (5.46%), and Lujiazui Subdistrict (4.88%). For PPSV23, the top three were Laogang Town (2.79%), Nicheng Town (2.01%), and Datuan Town (1.93%). Significant spatial clustering was observed for HZV and InfV. **Conclusions**: Vaccination coverage for HZV, InfV, and PPSV23 among adults aged ≥60 years in the Pudong New Area of Shanghai from 2020 to 2024 was generally low, with evident temporal variations and demographic and spatial disparities. Coverage differed by age group, gender, household registration status, and subdistricts or towns. These findings indicate that future interventions are still needed to increase vaccination coverage among older adults.

## 1. Introduction

Global demographic shifts have rendered population aging one of the most significant public health challenges of modern societies. By 2021, the global population reached approximately 7.9 billion, and more than 92% of countries worldwide experienced an increase in the proportion of individuals aged ≥65 years between 2000 and 2021 [1]. China, home to the world’s largest population of older adults, is among the fastest-aging countries globally. It is projected that by 2050, the number of individuals aged ≥65 years in China will reach 395 million, including 135 million aged ≥80 years [2]. With advancing age, the functional capacity of multiple organ systems gradually declines. This physiological decline, combined with age-related immunosenescence and the high prevalence of chronic comorbidities, significantly increases susceptibility to infectious diseases and may ultimately impair quality of life among older adults. Consequently, the health of older adults has become an increasingly prominent public health concern.

Evidence indicates that older adults are particularly susceptible to infectious diseases such as herpes zoster (HZ), influenza, and pneumococcal diseases. Once infected, they face a higher risk of severe illness, complications, and mortality [3]. HZ is caused by the reactivation of latent varicella-zoster virus when host immunity declines and is typically characterized by a unilateral dermatomal rash, often accompanied by postherpetic neuralgia. Advanced age is a well-established risk factor for HZ and its associated complications. HZ can occur at any age. However, its incidence increases markedly after 50 years of age and may reach approximately 50% by 80 years of age [4]. The incidence of HZ among individuals aged >65 years is approximately three times higher than that in younger populations [5], with mortality rates ranging from 0.0022 to 82.21 per 100,000 population across countries in Latin America [6]. Influenza is an acute respiratory infectious disease caused by the influenza virus and result in substantial morbidity and mortality each year, imposing a considerable burden on healthcare systems worldwide. Seasonal influenza is estimated to cause approximately 3–5 million severe cases and 290,000–650,000 deaths globally each year [7]. Although influenza affects individuals of all ages, the incidence is higher among those aged ≥60 years. A meta-analysis revealed that the global hospitalization rate for influenza among individuals aged ≥65 years (437 per 100,000) was more than five times higher than that among those <65 years (80 per 100,000) [8]. Streptococcus pneumoniae is the primary pathogen responsible for pneumococcal diseases such as otitis media, bacteremia, sepsis, meningitis, and community-acquired pneumonia, and predominantly affects children age <2 years and adults aged ≥65 years. In 2019, pneumococcal infections caused 829,000 deaths worldwide [9]. Moreover, advanced age is a major risk factor for severe pneumococcal diseases. A study conducted in the United States reported that the hospitalization rate for pneumococcal diseases among adults aged ≥65 years was 280 per 100,000 population, compared with 53 per 100,000 among adults aged 18–64 years, representing a 5.28-fold higher rate in the older age group [10], highlighting the substantially greater disease burden among older adults.

Effective vaccines against HZ, influenza, and pneumococcal diseases represent one of the most important strategies for reducing morbidity and mortality among adults aged ≥65 years [11]. The herpes zoster vaccine (HZV) has demonstrated substantial effectiveness in preventing HZ and reducing the economic burden among older adults [12]. A study conducted in Beijing, China, comparing the public health impact of HZV with no vaccination, estimated that large-scale implementation of HZV could prevent more than 430,000 cases of HZ, highlighting its potential population-level benefit [13]. The influenza vaccine (InfV) has been shown to effectively reduce the incidence of influenza, decrease the risk of severe complications, and lower influenza-related hospitalizations and mortality among older adults [3,14]. The 23-valent pneumococcal polysaccharide vaccine (PPSV23), which covers 23 common serotypes of streptococcus pneumoniae, has been widely used to reduce the risk of invasive pneumococcal diseases [15]. In addition, PPSV23 has been estimated to provide approximately 25% effectiveness in preventing against pneumococcal diseases [16]. However, despite the availability of these vaccines and their demonstrated effectiveness, the implementation of adult vaccination programs remains suboptimal globally, with coverage rates consistently falling short of targets [17,18,19].

In China, vaccination coverage among adults aged ≥60 years remains low. National cross-sectional studies have reported coverage rates of 3.8% for InfV [20]. Cheng et al. found that in 2022, the overall vaccination rates for PPSV23 and HZV across 31 provinces in China (excluding the Hong Kong Special Administrative Region, the Macao Special Administrative Region, and Taiwan Province) were 3.2% and 0.1%, respectively [21]. Moreover, substantial disparities in coverage exist across regions and population subgroups. Currently, most research data on adult vaccines in China are based on questionnaires and reviews, or analyses of single-vaccine administration. There is a lack of real-world vaccination data in studies evaluating multiple recommended vaccines for older adults. Therefore, this study utilizes real-world vaccination data for HZV, InfV, and PPSV23 among residents aged ≥60 years in the Pudong New Area of Shanghai from 2020 to 2024. This study aims to analyze the vaccination coverage and trends. Additionally, it aims to fill this part of the research content and provide a reference for optimizing immunization resource allocation and promoting vaccination efforts among older adults.

## 2. Materials and Methods

### 2.1. Vaccine Categories and Immunization Schedule

In China, HZV, InfV, and PPSV23 are categorized as non-National Immunization Program vaccines in most provinces, meaning that their administration is voluntary and primarily self-funded rather than universally government-financed. Two types of HZV are approved for use in China: the recombinant zoster vaccine (RZV) and the live attenuated zoster vaccine (ZVL). RZV is primarily recommended for adults aged ≥50 years as a two-dose series with an interval of 2–6 months, whereas ZVL is indicated for adults aged ≥40 years as a single dose. Approved InfV products include trivalent inactivated influenza vaccine, trivalent live attenuated influenza vaccine, and quadrivalent inactivated influenza vaccine. Inactivated influenza vaccines are further classified as split-virion vaccine or subunit vaccine based on their manufacturing process. Individuals aged ≥6 months are eligible to receive trivalent or quadrivalent inactivated influenza vaccines. Children aged ≥9 years and adults, including older adults, require one dose per influenza season. Currently, only PPSV23 is licensed for adult pneumococcal vaccination in China, with multiple domestic and imported products available on the market. It is recommended for susceptible individuals aged ≥2 years. The standard schedule is a single dose; if revaccination is indicated, it should be administered at least five years after the first dose, which is typically recommended for high-risk populations, such as immunocompromised individuals. Since 2013, Shanghai Municipality, China, has implemented public health programs offering one free dose of PPSV23 to eligible local residents aged 60 and older [22].

### 2.2. Data Sources

This study was conducted in the Pudong New Area, a large district located in the eastern part of Shanghai Municipality, China. As of the end of 2024, the resident population of Pudong had reached 5.78 million, accounting for one-fifth of the total population of Shanghai Municipality, and it comprises 36 subdistricts or towns. In this study, we collected and analyzed data on HZV, InfV, and PPSV23 administered to residents aged 60 years and older in Pudong New Area between 2020 and 2024. All vaccination records were obtained from the Shanghai Immunization Information System, a centralized electronic platform that captures individual-level immunization data. Each vaccination record contains detailed information, including the individual’s date of birth, gender, household registration status, the date of vaccination, and the specific type of vaccine administered, vaccination units, vaccination dates and vaccination doses. Population denominator data were obtained from the Pudong New Area Population Statistics Yearbooks.

### 2.3. Study Population

The inclusion criteria for this study were defined as follows: (1) individuals age ≥ 60 years; (2) permanent residents in the Pudong New Area of Shanghai; and (3) availability of electronic vaccination records for HZV, InfV, or PPSV23 in the Shanghai Immunization Information System. To ensure the quality and reliability of the data, individuals with missing or logically inconsistent vaccination data were excluded from the analysis. The age of each participant was calculated based on the date of birth. For the purpose of subgroup analysis, participants were categorized into five-year age groups: 60–64, 65–69, 70–74, 75–79, and ≥80 years.

### 2.4. Definitions

For HZV, which is not administered annually and has low overall coverage, individuals who received at least one dose during the study period were considered vaccinated. HZV coverage was calculated using the following formula:

(1) HZV coverage(%) = (number of individuals receiving ≥1 dose in the year)/(total population aged ≥60 years in the same year) × 100%

Given the annual administration of InfV, the number of doses actually administered in a given year equals the number of individuals vaccinated during that year. InfV coverage was calculated using the following formula:

(2) InfV coverage(%) = (number of individuals vaccinated in the year)/(total population aged ≥60 years in the same year) × 100%

For PPSV23, given the recommended 5-year revaccination interval and the generally low vaccination coverage, revaccination was not considered in this study. The number of doses actually administered in a given year equals the number of individuals vaccinated during that year. PPSV23 coverage was calculated using the following formula:

(3) PPSV23 coverage(%) = (number of individuals vaccinated in the year)/(total population aged ≥60 years in the same year) × 100%

### 2.5. Data Analysis

Data were organized and analyzed using R software (version 4.4.3). Categorical variables were summarized as counts and percentages. Differences in vaccination coverage between groups were assessed using the Chi-square test, and linear trends over time were evaluated using the Cochran–Armitage test. Spatial distribution patterns of vaccination coverage were examined using ArcMap software (version 10.2). Global spatial autocorrelation was assessed using *Moran’s I* statistic, with statistical significance determined by *Z*-scores and corresponding *p* value. All statistical tests were two-sided. The *p* value of less than 0.05 was considered statistically significant.

## 3. Results

### 3.1. Vaccination Coverage by Year

Between 2020 and 2024, a total of 26,227 doses of HZV, 198,373 doses of InfV, and 102,644 doses of PPSV23 were administered to adults aged ≥60 years in the Pudong New Area. Over this five-year period, the overall coverage rates were 0.23% for HZV, 3.12% for InfV, and 1.61% for PPSV23, respectively. Statistically significant temporal variations in coverage were observed for three vaccines (*p* < 0.001). The coverage of HZV peaked in 2023 (0.34%), while the lowest coverage was recorded in 2021 (0.17%). The coverage of InfV was highest in 2020 (3.94%) and the lowest in 2024 (2.41%). PPSV23 coverage reached its highest level in 2020 (3.21%) and its lowest level in 2022 (0.98%) (Table 1).

### 3.2. Vaccination Coverage by Demographic Characteristics

Vaccination coverage for all three vaccines differed significantly across age groups (*p* < 0.001). Specifically, HZV coverage was highest in the 70–74 age group (0.30%) and lowest among those aged ≥80 years (0.11%). InfV coverage peaked in the 75–79 age group (5.18%) and was lowest in the 60–64 age group (0.97%). PPSV23 coverage was highest in the 65–69 age group (2.15%) and lowest among those aged ≥80 years (1.06%) (Table 2).

When stratified by gender, HZV and InfV coverage was higher among females (0.27% and 3.39%, respectively) than among males (0.19% and 2.82%, respectively) (*p* < 0.001). In contrast, PPSV23 coverage was higher among males than females (*p* < 0.001) (Table 2).

When stratified by household registration type, individuals with local registration had higher coverage for HZV (0.02%), InfV (0.30%), and PPSV23 (0.45%) compared to those with non-local registration (0.01%, 0.21%, and 0.01%, respectively). The differences in coverage by registration type were statistically significant for all three vaccines (*p* < 0.001) (Table 2).

### 3.3. Vaccination Coverage by Subdistricts or Towns

**Figure 1:**
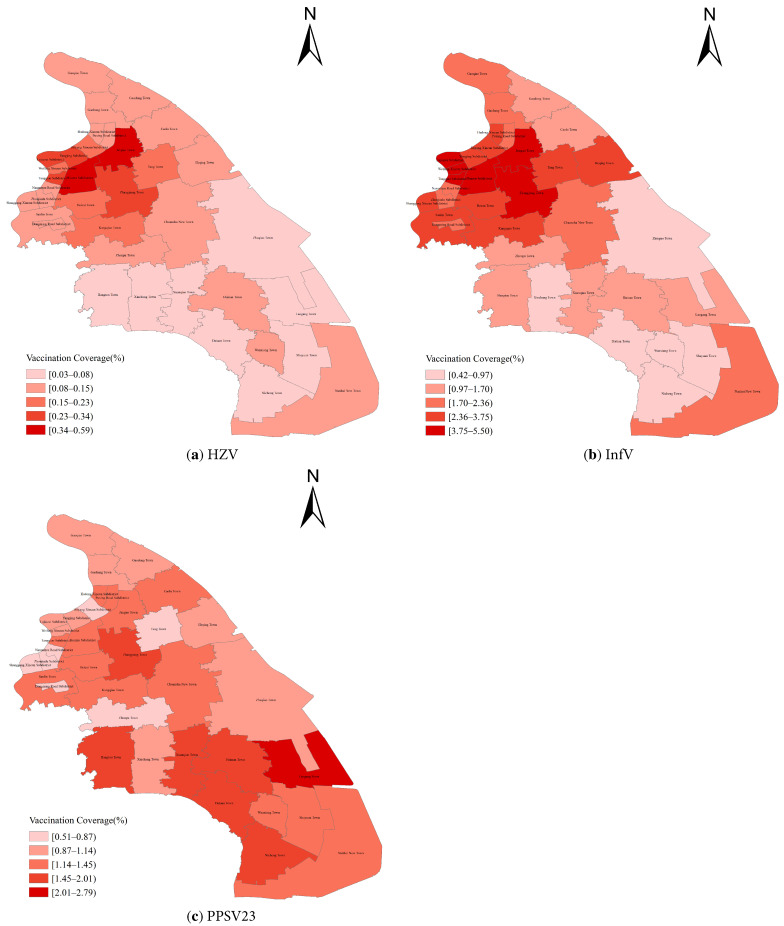
Vaccination coverage of three vaccines among adults aged ≥60 years across subdistricts or towns between 2020 and 2024.

The highest HZV coverage was observed in Jinqiao Town (0.59%), followed by Huamu Subdistrict (0.50%) and Lujiazui Subdistrict (0.34%) (Figure 1a). The highest InfV coverage was recorded in Tangqiao Subdistrict (5.50%), Huamu Subdistrict (5.46%), and Lujiazui Subdistrict (4.88%) (Figure 1b). PPSV23 coverage was highest in Laogang Town (2.79%), followed by Nicheng Town (2.01%) and Datuan Town (1.93%) (Figure 1c).

**Figure 2:**
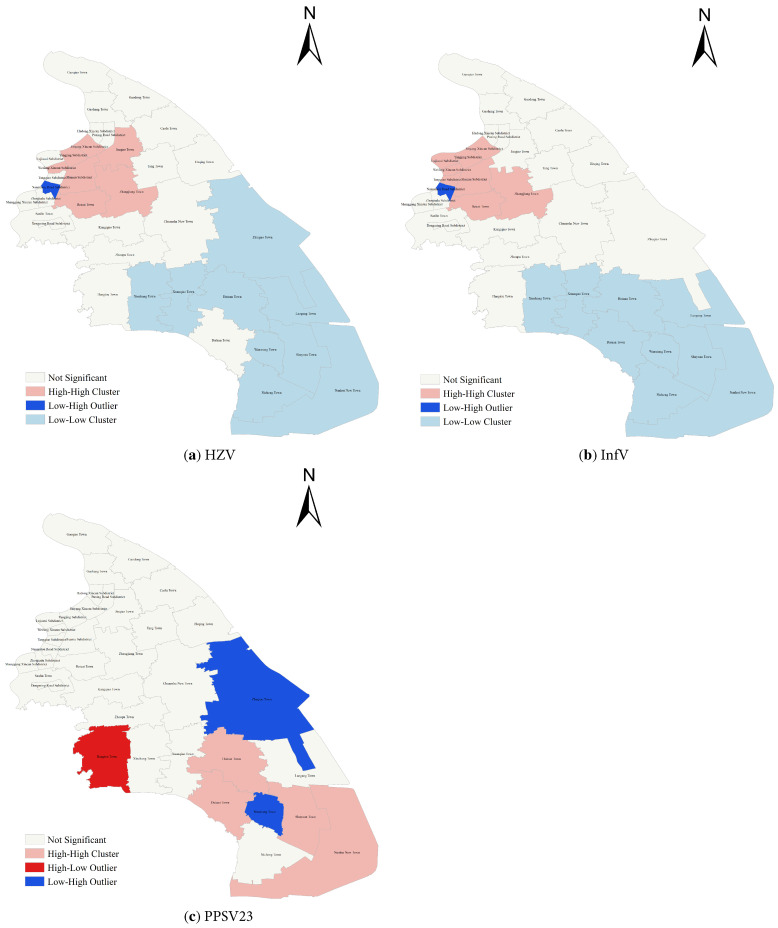
Local spatial autocorrelation of three vaccines among adults aged ≥60 years between 2020 and 2024.

Global spatial autocorrelation analysis conducted at the subdistrict or town level revealed significant spatial clustering of HZV coverage, with a *Moran’s I* of 0.458 (*Z* = 4.615, *p* < 0.001). Local spatial autocorrelation analysis further identified seven high-high clusters, one low-high cluster, and nine low-low clusters for HZV coverage (Figure 2a). Significant spatial clustering was also observed for InfV coverage, with a *Moran’s* *I* of 0.638 (*Z* = 5.967, *p* < 0.001). Eight high-high clusters, one low-high cluster, and nine low-low clusters were identified (Figure 2b). In contrast, no significant global spatial clustering was found for PPSV23 coverage, with a *Moran’s I* of 0.146 (*Z* = 1.646, *p* = 0.100). However, local spatial autocorrelation analysis identified four high-high clusters, two low-high clusters, and one high-low cluster (Figure 2c).

## 4. Discussion

Older adults represent a high-risk population for infectious diseases, and vaccination remains one of the most cost-effective preventive strategies. Currently, the key vaccines recommended for older adults include HZV, InfV, and PPSV23, which effectively prevent HZ, influenza, and pneumococcal diseases, respectively. The Chinese government and public health experts place high importance on vaccination against infectious diseases among the elderly. Multiple guidelines and expert consensus documents strongly recommend that older adults actively complete these three vaccines. Based on real-world vaccination data, this study systematically analyzed the coverage, temporal trends, and disparities across demographic groups and across subdistricts or towns for these three vaccines among individuals aged ≥60 years in the Pudong New Area of Shanghai from 2020 to 2024. The key findings of this study include the following: (1) overall coverage for HZV, InfV, and PPSV23 among older adults remained low and fluctuated over the five-year period; (2) age-stratified analysis revealed a “mid-elderly peak” pattern in vaccination coverage; (3) vaccination coverage differed by gender; (4) residents with local household registration had higher coverage for all three vaccines compared to those with non-local registration; and (5) substantial geographic disparities were observed at the subdistrict and town levels, with significant spatial clustering observed for HZV and InfV coverage.

The overall coverage for HZV, InfV, and PPSV23 among older adults in the Pudong New Area of Shanghai were 0.23%, 3.12%, and 1.61%, respectively. The HZV coverage was comparable to that reported among older adults in Beijing from 2020 to 2022, where vaccination rates were 0.11%, 0.25%, and 0.54% respectively [21]. However, it was markedly lower than the coverage reported in the United States in 2018 (41.1%) [23]. Several provinces and municipalities in China, including Beijing and Zhejiang, have implemented free InfV vaccination programs, resulting in significantly higher coverage compared to regions such as Shanghai that have not yet adopted such policies. Among adults aged ≥60 years in Beijing Municipalities, the InfV coverage from 2019 to 2023 were 16.06%, 18.83%, 15.03%, and 14.14%, respectively. In Zhejiang Province, the corresponding rates were 7.32%, 13.50%, 21.39%, and 20.41%, respectively [21]. The InfV coverage observed in our study was slightly higher than rates reported in Tianjin (3.05%) and Hubei (2.64%) [21], which also lack free vaccination programs, yet remained far below the United States coverage of 70% [24]. The PPSV23 coverage was substantially lower than rates reported in the United States (69.0%) and South Korea (54.5%) [25]. These findings collectively underscore the urgent need to improve vaccination coverage among older adults in China through targeted public health strategies and policy interventions.

Currently, China lacks a national free HZV policy. A previous study analyzed respondents’ willingness to receive the HZV under different payment scenarios. The results showed that if HZV were included in basic medical insurance, the vaccination willingness rate would increase substantially from 16.57% to 72.25% [26]. This finding indicated that vaccine cost remains a significant barrier to HZV uptake among older adults. Additionally, factors contributing to the low HZV vaccination coverage include insufficient awareness and knowledge of the vaccine among the elderly. In a cross-sectional survey conducted in Ningbo, China, 48.2% of respondents reported never having heard of HZV [27]. Therefore, high out-of-pocket cost, coupled with limited awareness among older adults, may contribute to pronounced vaccine hesitancy [28]. Targeted health education and public awareness campaigns are needed to improve understanding of HZ and the protective benefits of vaccination. International experience shows that active recall strategies also play a significant role in improving vaccination coverage. A study from Italy found that from 2017 to 2023, this measure increased HZV vaccination coverage from 44.4% to 54.9% [29]. At present, more than 40% of countries or regions worldwide have incorporated influenza vaccination for high-risk populations, such as children and older adults, into their national immunization programs. However, China has not yet adopted such a policy at the national level. Previous research indicated that 31.5% of older adults were more likely to get vaccinated when the InfV costs were covered by medical insurance [30]. Therefore, considering the economic burden associated with influenza, more standardized and regular reimbursement policies are needed in the future to ensure sustained immunization coverage and public health. A systematic review and meta-analysis found that hospitals, as high-contact settings for older adults, present important opportunities for vaccination. When clinicians actively reminded patients about vaccination, InfV coverage increased from 10% to 36%. For PPSV23, the coverage increase was even more pronounced, rising from 23% to 62% [31]. Healthcare workers are typically an important source of vaccination information for older adults, and recommendations from healthcare professionals can substantially increase their willingness to be vaccinated. Under the current family doctor system in China, it is recommended that contracted family physicians provide professional vaccination assessments and recommendations for older adults under their care.

Vaccination coverage for all three vaccines fluctuated across the study period, with higher coverage observed in 2020 and 2023, exhibiting an overall decline–rise–decline trend. This temporal pattern differs from trends observed in other countries and regions. For example, data from Bavaria, Germany, indicated a decline in InfV coverage from 2020 to 2021, while HZV and PPSV23 coverage continued to rise during the same period [32]. In the United States, HZV coverage among older adults has increased steadily since 2008, reaching 42.2% in 2023 [33]. In the present study, HZV coverage peaked in 2023 at 0.34%. This peak may have been facilitated by the market entry of ZVL, which is priced lower than RZV and may have temporarily stimulated demand among cost-sensitive older adults. Furthermore, InfV and PPSV23 coverage were highest in 2020 (3.94% and 3.21%, respectively). Multiple studies have indicated that during the COVID-19 pandemic, due to the similar symptoms of influenza and COVID-19, residents became more aware of the severity of respiratory infectious diseases. This heightened awareness led to increased interest in pursuing health-promoting behaviors, such as receiving InfV and PPSV23 [34,35,36]. Meanwhile, public health messaging during the pandemic shaped the general public’s perceived susceptibility to viral infection and was linked to greater motivation for InfV in 2020 [37]. The subsequent diversion of healthcare resources to pandemic control may have constrained the promotion of non-COVID vaccines, contributing to coverage fluctuations in later years [38].

Age-stratified analysis revealed that coverage for all three vaccines initially increased with advancing age before declining, reflecting a “mid-elderly peak” pattern. Specifically, HZV coverage was highest among individuals in the 70–74 age group, PPSV23 coverage peaked in the 65–69 age group, and InfV coverage was highest in the 75–79 age group. These trends were consistent with previous studies, where coverage among individuals in the 70–79 age group exceeded that of other age groups [39]. This observed pattern may reflect increased awareness of disease risks and vaccine benefits with advancing age, coupled with greater influence from physician recommendations and family encouragement [40]. A key finding from Shandong, China, was that 91.5% of those unwilling to be vaccinated expressed concerns regarding vaccine quality [41]. Furthermore, this limited vaccine confidence among healthcare workers was found to be a contributing factor to lower vaccination coverage among the elderly. Hence, Strengthening healthcare workers’ knowledge of vaccine safety may enhance vaccination willingness and uptake among older adults.

Gender-stratified analysis indicated that HZV and InfV coverage was higher among females, while PPSV23 coverage was higher among males. Females generally engage more frequently in healthcare-seeking behaviors and routine health check-ups, displaying higher levels of health awareness [42,43]. Additionally, females exhibit a higher incidence of HZ [44], which may drive greater demand for HZV among this population. Males are more likely to engage in risk behaviors such as smoking, which increases susceptibility to pneumococcal diseases [45] and may influence healthcare providers to preferentially recommend PPSV23 for male patients. Beyond policy-related factors, socioeconomic status, social engagement, and living conditions also impact vaccination uptake, which may explain the consistently higher coverage among residents with local household registration. Spatial distribution analysis revealed significant spatial autocorrelation for HZV and InfV coverage. High-high clusters were predominantly located in the more economically developed northern areas of Pudong, including Jinqiao Town, Huamu Subdistrict, Lujiazui Subdistrict, and Tangqiao Subdistrict. Older adults in these areas likely have better access to health information and greater financial capacity to afford self-paid vaccines, leading to a higher propensity for proactive vaccination [46]. In contrast, the spatial autocorrelation of PPSV23 was not statistically significant, suggesting that overall coverage was randomly distributed across the study area. High-high clusters for PPSV23 were mainly concentrated in the less economically developed southern areas, such as Laogang Town, Nicheng Town, and Datuan Town. This pattern suggests that the free PPSV23 vaccination policy may play a key role in promoting uptake by reducing financial barriers in these populations [47]. Collectively, these findings indicate substantial regional heterogeneity in vaccine uptake and highlight the importance of developing differentiated, area-specific vaccination promotion strategies. Particular emphasis should be placed on strengthening community-based health education and outreach in subdistricts or towns with lower coverage to improve overall vaccination uptake among older adults.

This study has several limitations. First, the study was based on data collected from the Pudong New Area, Shanghai, China. The results may be influenced by local variations in healthcare resources, vaccine supply, and policies, which limits the generalizability of the findings. Second, defining HZV coverage as receipt of at least one dose may overestimate the proportion of individuals with effective protection, especially for RZV, which requires two doses series to achieve optimal immunogenicity. Third, the InfV and PPSV23 coverage estimates in this study reflect dose-based annual uptake rather than cumulative individual-level coverage. Fourth, the absence of individual-level data on socioeconomic status, health behaviors (e.g., smoking, medical history, healthcare utilization) precluded a more detailed analysis of factors influencing vaccination uptake. Future research will incorporate questionnaire surveys to collect comprehensive individual-level information, thereby enabling a more refined analysis of the determinants of vaccination coverage and informing targeted intervention strategies.

## 5. Conclusions

Vaccination coverage for HZV, InfV, and PPSV23 among residents aged ≥60 years in Shanghai remains suboptimal, with notable disparities observed across age groups, gender, and subdistricts or towns. Vaccination efforts targeting older adults should continue to receive high priority in public health policy and program planning.

## Figures and Tables

**Table 1:** Vaccination coverage of three vaccines among adults aged ≥60 years between 2020 and 2024.

Vaccine Type	2020	2021	2022	2023	2024	Total *	*χ* ^2^	*p*
HZV	5217 (0.22)	4161 (0.17)	4325 (0.19)	7775 (0.34)	4749 (0.23)	26,227 (0.23)	221.900	<0.001
InfV	48,488 (3.94)	35,885 (2.88)	33,471 (2.73)	51,032 (3.90)	29,497 (2.41)	198,373 (3.12)	1726.100	<0.001
PPSV23	39,525 (3.21)	12,584 (1.01)	12,065 (0.98)	23,179 (1.77)	15,291 (1.13)	102,644 (1.61)	8263.200	<0.001

Note: Data are the number of vaccine doses administered (% of vaccination coverage). * Total number of vaccine doses administered (annual average vaccination coverage).

**Table 2:** Vaccination coverage of three vaccines by demographic characteristics among adults aged ≥60 years between 2020 and 2024.

Basic Characteristics	HZV	InfV	PPSV23
Age Group			
60–64	4963 (0.17)	16,914 (0.97)	20,807 (1.20)
65–69	9550 (0.29)	56,568 (3.12)	39,016 (2.15)
70–74	7035 (0.30)	58,816 (4.56)	22,301 (1.73)
75–79	3195 (0.25)	36,521 (5.18)	11,906 (1.69)
≥80	1484 (0.11)	29,554 (3.64)	8614 (1.06)
*χ* ^2^	20.817	24,792.000	3690.100
*p*	<0.001	<0.001	<0.001
Gender			
Male	15,940 (0.19)	86,040 (2.82)	49,134 (3.11)
Female	10,287 (0.27)	112,333 (3.39)	53,510 (3.06)
*χ* ^2^	430.650	1706.000	11.549
*p*	<0.001	<0.001	<0.001
Household Registration			
Local	17,745 (0.02)	153,630 (0.30)	96,573 (0.45)
Non-local	8482 (0.01)	44,743 (0.21)	6071 (0.01)
*χ* ^2^	62.792	4930.807	203,538.100
*p*	<0.001	<0.001	<0.001

Note: Data are the number of vaccine doses administered (% of vaccination coverage).

## Data Availability

Restrictions apply to the availability of these data, which were collected from the Shanghai Immunization Information System. Data are available from the authors with permission from the Shanghai Pudong New Area Center for Disease Control and Prevention (Shanghai Pudong New Area Health Supervision Institute).

## Abbreviations

HZ	Herpes Zoster
HZV	Herpes Zoster Vaccine
InfV	Influenza Vaccine
PPSV23	23-valent Pneumococcal Polysaccharide Vaccine
RZV	Recombinant Zoster Vaccine
ZVL	Live Attenuated Zoster Vaccine
